# The Use of Coupled Plasma Filtration Adsorption in Traumatic Rhabdomyolysis

**DOI:** 10.1155/2017/5764961

**Published:** 2017-03-19

**Authors:** Mario Pezzi, Silvia Renda, Anna Maria Giglio, Anna Maria Scozzafava, Simona Paola Tiburzi, Patrizia Casella, Fabrizio Iannelli, Mario Verre

**Affiliations:** ^1^Anaesthesia and Intensive Care Unit, General Hospital “Pugliese-Ciaccio”, Viale Pio X, 88100 Catanzaro, Italy; ^2^Bellco, 41037 Mirandola, Italy

## Abstract

Severe musculoskeletal injuries induce the release of sarcoplasmic elements such as muscle enzymes, potassium, and myoglobin in the systemic circulation. The circulating myoglobin damages the glomerulus and renal tubules. Conventional haemodialysis is not able to remove myoglobin, due to its high molecular weight (17,8 kilodaltons [kDa]). We treated four traumatic rhabdomyolysis patients with Coupled Plasma Filtration Adsorption (CPFA) in order to remove myoglobin followed by 14 hours of Continuous Veno-Venous Hemofiltration (CVVH). During the treatment, all patients showed clinical improvement with a decrease in muscular (creatine kinase [CK] and myoglobin) and renal (creatinine and potassium) damage indices. One patient, in spite of full renal recovery, died of cerebral haemorrhage on the 26th day of hospital stay.

## 1. Introduction

Musculoskeletal damage in trauma patients includes several clinical presentations [1].

Among the various forms of rhabdomyolysis, the common pathophysiologic element is a rise in intracellular ionized calcium. The loss of transcellular calcium gradient causes a series of events which lead to cell death [2].

If rhabdomyolysis is suspected it is important to evaluate creatine kinase (CK) and myoglobin blood levels, since they represent the most sensitive markers of muscle injury. Changes in creatinine, potassium, sodium, blood urea nitrogen, total and ionized calcium, magnesium, phosphate, uric acid, albumin, and lactate blood levels may also be present. Other important investigations include acid base balance evaluation, blood cell count, and coagulation tests.

The enzyme creatine kinase is present in both skeletal and cardiac muscles in its two isoforms, MM and MB. In the skeletal muscle, MB isoenzyme accounts for 90% of total CK and its dosage is specific for muscular disorders. Normal CK blood levels are up to 170 U/L: after extended muscle injury, this value undergoes a more than fivefold increase between 2 and 12 hours after the onset of the lesion, reaches a peak (up to 100000 U/L) within 24 to 72 hours, and then decreases within 3–5 days after the resolution of the muscular lesion [3].

A fivefold elevation of serum CK in absence of cardiac or neurological disorders is diagnostic of rhabdomyolysis, and a value of more than 5000 U/L can be associated with the development of renal failure [4]. CK serum levels remain elevated longer than myoglobin, due to its relatively slow plasma clearance rate (1.5 days).

Myoglobin is a small protein with a very low hematic concentration in physiological conditions; it is composed of 153 amino acids with a single heme prosthetic group, with a molecular weight of 17,8 kDa. Myoglobin accounts for 1–3% of muscle mass dry weight and its function is to bind oxygen and facilitate its transport within muscle cells, which work under low oxygen tension conditions. Myoglobin serum levels increase within 1 hour after a musculoskeletal injury and return to baseline within 1 to 6 hours after the resolution of the lesion; its metabolism occurs by glomerular filtration, absorption in the proximal tubule by endocytosis, and proteolysis.

When a muscle injury takes place, free myoglobin enters the bloodstream and largely binds to haptoglobin and *α*2 globulins. Myoglobin removal from the circulation is due to the reticuloendothelial system, but in rhabdomyolysis free myoglobin concentration exceeds its purifying capability [5].

The main consequence of a massive release of myoglobin in the systemic circulation is acute renal failure. The relationship between myoglobin blood levels and renal injury was demonstrated by Kasaoka et al. in a study including 30 rhabdomyolysis patients: according to these authors, the peak serum levels of CK and myoglobin were significantly higher in the group with acute renal failure than in the group with preserved renal function [6].

This phenomenon is linked to the degree of acidity of urine which is, in turn, correlated with hypovolemia. Myoglobin levels exceeding 15000 ng/mL are significantly related to the development of acute renal failure and the need for haemodialysis [7].

Correction of hypovolemia with an early and aggressive fluid resuscitation has proven to be the most important factor in preventing the development of renal failure. During the first 72 hours more than 10 litres of fluids per day may be needed, so strict monitoring of diuresis and central venous pressure is essential to avoid volume overload and pulmonary oedema. The goal should be a urine flow rate of more than 2-3 mL/kg/h; it is advisable to avoid solutions containing potassium [8]. The use of loop diuretics and mannitol may be beneficial, while the role of urine alkalinization remains controversial [9].

From November 2013 to December 2015, four patients with rhabdomyolysis came to our attention: they all presented raised serum CK and myoglobin levels and were promptly treated with fluid replacement, urine alkalinization, and forced diuresis, in order to prevent myoglobin-induced renal damage.

Since low-flow haemodialysis does not allow the elimination of substances with a molecular weight exceeding 5 kDa, the removal of high molecular weight proteins such as myoglobin requires high volume hemodiafiltration techniques [10].

To foster the removal of myoglobin, we used Coupled Plasma Filtration Adsorption (CPFA) associated with Continuous Veno-Venous Hemofiltration (CVVH). The patients were subjected to extracorporeal treatment with CPFA for 10 hours followed by CVVH for 14 hours. Two patients were subjected to three treatments in 72 hours and two patients were subjected to four treatments within 96 hours.

After control and correction of haemoglobin, platelets and antithrombin III levels, and coagulation profile, anticoagulation was obtained with a priming dose of 15000 U in 4 litres of washing liquid and a maintenance dose of 5–10 U/kg/h of unfractioned heparin, with monitoring of activated Partial Thromboplastin Time (aPTT) and Activated Clotting Time (ACT) every 4 hours. Target was aPTT (not > 60 sec) and ACT (140–200 sec). For automatic priming, the infusion liquid is used for the filling and washing. This condition predisposes the apparatus not only to the priming but also to the patient's treatment.

**Figure 1:**
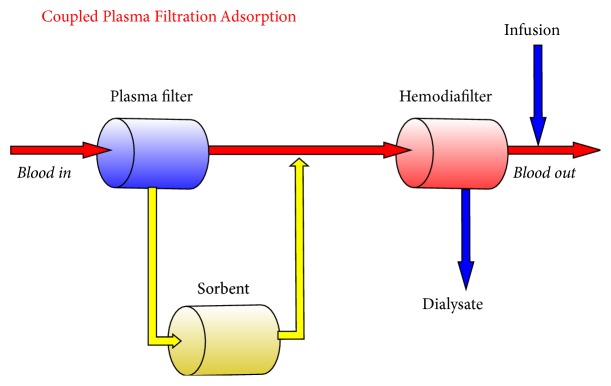


CPFA is an extracorporeal therapy which uses a plasma filter to separate plasma from blood, allowing the separated plasma to pass through an adsorbent cartridge for nonspecific removal of several mediators. After purification, plasma is returned to blood, which can then pass through a hemofilter for further purification by means of conventional haemodialysis, hemofiltration, or hemodiafiltration in case of acute renal failure (Figure 1). The main therapeutic goal of CPFA is to hit the excess of pro- and anti-inflammatory mediators, in order to reestablish a normal immune function [11].

In CPFA a synthetic resin made of styrene-divinylbenzene is used which interacts with the hydrophobic sites on the molecules: this type of resin is used in various processes ranging from chromatography purification to industrial food, beverage, and pharmaceutical products. This resin is suitable for extracorporeal applications for its high homogeneity, the good performance pressure-flow, and excellent mechanical and chemical stability. In addition to its mechanical properties, the choice of this particular resin was based on various other factors related to its ability to adsorb a wide variety of inflammatory mediators and its adsorption characteristics at different flow rates. The maximum of efficacy is obtained especially in case of substances with high-medium molecular weight, like myoglobin (about 18 kDa).

CPFA consists of a four-pump modular device (Lynda, Bellco®, Mirandola, Italy) with a plasma filter (0.45 m^2^ polyethersulfone with an approximate cut-off of 800 kDa), a nonselective hydrophobic resin cartridge (140 mL) with a surface of approximately 700 m^2^/g, and a synthetic, high-permeability hemofilter (1.4 m^2^ polyethersulfone) which allows the application of convectional exchange to the whole blood once it is reconstituted in postdilution [12].

The distinctive aspect of CPFA is the application of the sorbent to the plasma rather than the whole blood. This feature brings important benefits: the lower flow of plasma allows a prolonged contact with the sorbent and reduces biocompatibility issues [12].

We used the blood flow (Qb) values that were between 200 and 250 mL/min, and the amount of reinfusion solution (Qinf) used was 35 mL/kg/h. The plasma flow value (Qs) was 15% compared to the prescribed blood flow.

In the intervals between treatments with CPFA all patients were treated with CVVH. Blood flow (Qb) between 150 and 220 mL/min has been used according to the set values of ultrafiltration, in order to obtain a fraction of filtration (FF) <20%. The amount of reinfusion solution (QR) used was 35 mL/kg/h, with a share of 30% infusion in predilution.

To make the CVVH with Lynda device Bellco is necessary to exclude the plasma filter and the sorbent cartridge and use only the hemofilter. It allows high volumes of ultrafiltrate using the filling liquid that can be administered before or after the filter. The pump ensures adequate blood flow to maintain the proportion of ultrafiltrate required.

## 2. Clinical Cases

### 2.1. Male, Aged 19

The patient presented with a gunshot wound in his left lower limb, haemorrhagic shock, muscular disintegration, left popliteal artery occlusion, and multiple venous lesions; furthermore, there was another gunshot wound in the skull with left temporal bone fracture and a retained bullet in proximity to the 5th cervical vertebral body.

The patient underwent vascular surgery after approximately 8 hours following the traumatic event. Arterial vascularization was restored, fasciotomy of the left lower limb was performed, and continuous aspiration wound dressing was placed.

During the surgery, 8 units of packed red blood cells, 4 units of fresh frozen plasma, and 4 units of platelets were administered; in the following 24 hours fluid replacement was initiated under continuous monitoring of central venous pressure: 5% glucose (3000 mL), 0,9% normal saline solution (3000 mL), and 8,4% sodium bicarbonate for urine alkalinization (500 mL) were infused. The urine output was approximately 200 mL/h.

After the surgery the patient was transferred to the Intensive Care Unit, intubated, and under mechanical ventilation and inotropic support with Dopamine.

48 hours after the traumatic event, a further increase in CK (14790 U/L) and myoglobin (7881 ng/mL) levels occurred; the creatinine value was 1,1 mg/dL, while the potassium value was 5,2 mEq/L.

At this point Continuous Renal Replacement Therapy (CRRT) was initiated, alternating CPFA during the first 10 hours and CVVH for the remaining 14 hours.

Valid, spontaneous diuresis was present throughout the treatment, for a total of 72 hours.

**Figure 2:**
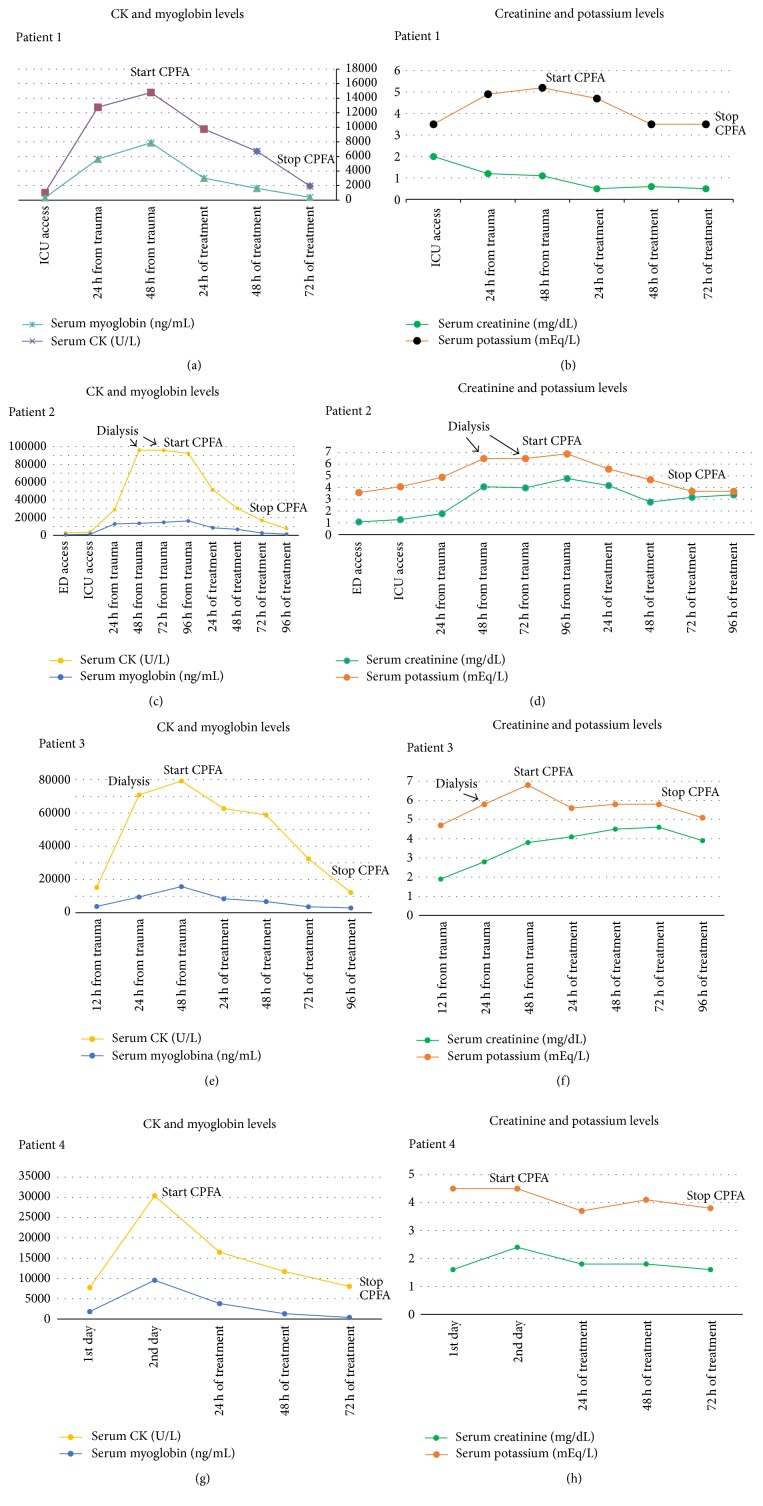


Given the positive evolution of clinical course and laboratory values, CRRT was stopped. On the 9th day the patient was transferred to the Vascular Surgery Department for continuation of hospital care (Table 1, Figures 2(a) and 2(b)).

### 2.2. Male, Aged 55

The patient was brought to the Emergency Department after a road trauma involving a heavy vehicle, with crushing of pelvis and lower limbs. He presented haemorrhagic shock due to multiple pelvic girdle fractures, traumatic section of urethra, retroperitoneal hematoma, left femoral fracture, L2 to L5 vertebral body fractures, and crush injury of the right lower limb.

After suprapubic catheterization, the patient underwent external fixation of pelvic fractures and reduction of left femoral fracture.

Fasciotomy with confection of a continuous aspiration dressing was performed on the right lower limb. 10 units of packed red blood cells, 4 units of fresh frozen plasma, and 2 units of platelets were administered.

During the next 24 hours, under continuous monitoring of central venous pressure, 4000 mL of 5% glucose, 4000 mL of 0,9% normal saline, and 1000 mL of 8,4% sodium bicarbonate were infused; the patient also needed inotropic support with continuous infusion of Adrenaline and Dopamine.

Given the onset of acute renal failure, two sessions of haemodialysis were carried out at 48 and 72 hours after the trauma. At 96 hours, given the rising levels of serum CK and myoglobin, CRRT was commenced alternating 10 hours of CPFA with 14 hours of CVVH.

The treatment lasted 96 hours; then it was suspended due to clinical improvement and decrease of muscular and renal damage indices.

During the following days a further improvement was observed, and after one month of ICU stay the patient was transferred to a recovery department, from which he was then discharged (Table 1, Figures 2(c) and 2(d)).

### 2.3. Male, Aged 33

The patient presented crush injury of both lower limbs due to overturn of a quad bike, with bilateral femoral and tibial fractures, right femoral artery section, and extended muscular damage of both lower limbs.

He was initially admitted to a spoke hospital where traction was applied to lower extremities, and after approximately 12 hours he was transferred to our centre for definitive treatment of bone, muscle, and vascular injuries.

Arterial revascularization of the right lower limb, reduction of fractures and fasciotomy of both lower extremities were performed. During the surgery the patient received 10 units of packed red blood cells, 5 units of fresh frozen plasma, and 2 units of platelets.

After the surgery the patient was brought to the ICU where he remained intubated and under mechanical ventilation. During the following 24 hours, 5000 mL of 5% glucose, 5000 mL of 0,9% normal saline, and 1000 mL of 8,4% sodium bicarbonate were administered under continuous central venous pressure monitoring. The patient also required inotropic support with continuous infusion of Adrenaline and Dopamine.

Six hours after the surgical interventions the right lower limb became ischaemic again and was amputated. The left lower limb and the stump of the amputated extremity were treated with a sealed continuous aspiration dressing.

24 hours after the traumatic injury the patient became anuric, so a three-hour haemodialysis session was performed.

Since anuria persisted at 48 hours and laboratory exams showed further deterioration of renal function, CRRT therapy was started with CPFA for 10 hours and CVVH for 14 hours.

Given the improvement trend, CRRT was suspended after 96 hours of treatment and haemodialysis was restarted with a two-hour treatment on alternate days for three more days. During the following days renal function was restored completely and both respiratory and hemodynamic parameters normalized. The continuous aspiration dressing was kept in site. On the 26th day the patient suddenly died due to cerebral haemorrhage.

Enoxaparin 4000 IU was administered twice daily to the patient throughout the period of hospitalization. The autopsy showed cerebral haemorrhage with ventricular flooding and diffuse cerebral edema. It is not inconceivable that the enoxaparin had a causal role in the genesis of cerebral haemorrhage (Table 1, Figures 2(e) and 2(f)).

### 2.4. Male, Aged 25

The patient was transported to our Emergency Department after a 12-meter fall: he presented with multiple rib fractures, hemoperitoneum due to spleen rupture, multiple pelvic fractures, right femoral fracture, right ulnar fracture, extended muscular injury of the right upper and lower limb, and haemorrhagic shock.

He immediately underwent splenectomy and bilateral chest drain positioning. During the surgery 6 units of packed red blood cells, 4 units of fresh frozen plasma, and 2 units of platelets were given. Closed reduction of limb fractures was also performed.

During the following 24 hours the patient received 4000 mL of 5% glucose, 4000 mL of 0,9% normal saline, and 1000 mL of 8,4% sodium bicarbonate, with close monitoring of central venous pressure. Due to hemodynamic instability, inotropic support with continuous infusion of Dopamine was carried out.

In the ICU the patient underwent pharmacological sedation, several blood and hemoderivatives transfusions, mechanical ventilation, and antibiotic therapy. On the 5th day a new surgery was performed for internal fixation of femoral and elbow fractures and external fixation of pelvic fractures.

On the 1st day of stay, after splenectomy, laboratory tests showed a creatinine level of 1,6 mg/dL, a potassium level of 4,5 mEq/L, and CK and myoglobin values of 7780 U/L and 1830 ng/mL, respectively.

On the 2nd day a sharp rise in muscle damage indices was observed, with CK levels of 30389 U/L and myoglobin levels of 9562 ng/mL and concomitant increase of creatinine value (2,4 mg/dL), while potassium levels remained stable (4,5 mEq/L).

CRRT was started, alternating 10 hours of CPFA with 14 hours of CVVH. The treatment was carried out for a total of 72 hours with continuous improvement of serum CK and myoglobin levels and renal function parameters.

After 40 days of ICU stay the patient was transferred to a rehabilitation centre in good clinical conditions (Table 1, Figures 2(g) and 2(h)).

## 3. Discussion

Acute renal failure due to myoglobinuria is the most dangerous complication of both traumatic and nontraumatic rhabdomyolysis and can be life-threatening [13].

When acute renal injury is severe enough to produce refractory hyperkalemia, metabolic acidosis, or volume overload, renal replacement therapy is indicated, mainly by intermittent haemodialysis which is able to rapidly and efficiently correct electrolyte disturbances.

Nevertheless, conventional haemodialysis is not able to remove myoglobin properly due to its high molecular weight and is generally started for renal indications: so, given its pathogenetic role in the development of acute renal injury during rhabdomyolysis, preventive extracorporeal elimination of myoglobin has been studied [9].

New “super high-flux” membranes for haemodialysis have been developed with a high cut-off pore size allowing efficient removal of middle and large size uremic toxin that cannot be removed by conventional dialysis membranes. The recent availability of a new generation of haemodialysis membranes with molecular weight cut-offs closer to that of the native kidney (65 kDa) has led to great benefits in several different clinical settings. These membranes have shown efficient removal of myoglobin in patients with rhabdomyolysis [14].

Effective removal of myoglobin by haemodialysis using a single-pass batch system and a high-flux polysulfone dialyzator (1,8 m^2^ surface), able to remove substances with a molecular weight as high as 30 kDa, has been reported [15].

The use of a large pore, high cut-off membrane for haemodialysis has been described [16].

During CVVH, or hemodiafiltration, the use of high-flux, high volume (convection) filters showed good effectiveness in removing myoglobin in acute rhabdomyolysis [17].

The membrane and technique used in the process of separation of myoglobin are crucial to the success of therapy. Since standard membranes are substantially impenetrable to myoglobin, high-flux membranes should be used [18].

Experience with the use of CPFA in rhabdomyolysis, especially posttraumatic, is very limited.

Lai et al. reported the successful use of CPFA in two kidney transplant patients with post-nephrolithotomy septic shock and severe rhabdomyolysis of unknown origin [19].

Ronco suggests the use of absorption on whole blood directly or CPFA, in which the patient's plasma is reinfused once regenerated by passage through a sorbent cartridge [18].

Instead, many reports about the use of plasmapheresis exist [20].

Attempts to use plasmapheresis have resulted in higher sieving coefficients, but the final clearance is minimal because of the limitations imposed by low volume exchanges.

The affinity between plasmapheresis and CPFA is well known: these two techniques are both able to eliminate molecules with a molecular weight exceeding 15 kDa.

While plasmapheresis implies the separation of the cellular components of blood from plasma, which can be then eliminated or further filtrated (selective apheresis), CPFA consists of a combination of apheresis and haemodialysis: this technique, in fact, combines plasma separation with adsorption and postdilutional haemodialysis. The circuit includes a plasma filter (800 kDa cut-off) which initially separates plasma from the cellular components of blood and a cartridge containing a hydrophobic resin (styrene-divinylbenzene, mean diameter of single grain 75 microns, diameter of pores 30 nm, and surface area 700 m^2^/g) on which plasma coming from the plasma filter is perfused at a rate of 30–40 mL/min; finally, this purified plasma passes through a hemofilter, together with the cellular components coming from the plasma filter.

The hydrophobic resin cartridge allows the removal of toxic molecules (cytokines, bilirubin, and myoglobin) without loss of nutrients such as albumin [21].

Throughout the treatment with CPFA, our patients showed a positive trend of clinical improvement with a decrease in muscular (CK and myoglobin) and renal (creatinine and potassium) damage indices.

In all patients, we began treatment with CPFA after standard therapy, intravenous fluids, urine alkalinization, surgical procedures, and diuretics. In two patients with acute kidney injury, the treatment CPFA was preceded by haemodialysis. Despite standard therapy CK and myoglobin values were increasing rapidly. In all cases already after the first treatment we observed a decrease of the values of CK and myoglobin. If CK and myoglobin values were down after the standard treatment we would not have started CPFA.

CK and myoglobin values continued to fall in the interval between treatments with CPFA. CVVH was used during the washout periods. The decrease of the blood values of CK and myoglobin was slower after the treatments with CVVH compared to the values found after treatment with CPFA (Table 1).

## 4. Conclusions

We treated 4 traumatic rhabdomyolysis patients with CPFA. CPFA was combined with CVVH. All patients showed a significant reduction in CK and myoglobin blood levels, along with an improvement in renal function. During the treatment, all patients maintained good respiratory and hemodynamic stability and no complications were seen.

Three patients survived and completely recovered after a rehabilitation period. One patient suddenly died on the 26th day for reasons not directly related to muscle injury or renal failure (cerebral haemorrhage).

Our experience was positive but remains limited: in order to assess the real effectiveness of CPFA in traumatic rhabdomyolysis, a multicentric study involving ICUs where major traumas are treated may be of interest. It would be useful to study with an assessment of the hourly changes in serum CK and myoglobin values during treatment in order to confirm the effectiveness of the CPFA to remove myoglobin from the blood.

## Figures and Tables

**Table 1:** 

	First treatment CPFA 10 h	Second treatment CPFA 10 h	Third treatment CPFA 10 h	Fourth treatment CPFA 10 h
*Patient 1*				
*Blood flow *Qb, mL/min	220	200	200	
Total plasma mL	7.500	7.000	7.250	
Plasma filtration flow rate, QS%	15	15	15	
Total dose of heparin I.U.	1.250	1.250	1.250	
*Reinfusion solution *Qinf, mL/min	35	35	35	
CK value U/L before CPFA	14.790	9.763	6.727	
CK value U/L after CPFA	10.725	7.659	2.512	
Myoglobin value ng/mL before CPFA	7.881	3.625	1.634	
Myoglobin value ng/mL after CPFA	4.412	1.829	823	

*Patient 2*				
*Blood flow *Qb, mL/min	*250*	*250*	*220*	*220*
Total plasma mL	*9.000*	*8.500*	*8.500*	*8.200*
Plasma filtration flow rate, QS%	*15*	*15*	*15*	*15*
Total dose of heparin I.U.	1.250	1.250	1.250	1.250
*Reinfusion solution *Qinf, mL/min	*35*	*35*	*35*	*35*
CK value U/L before CPFA	92.373	51.290	30.664	16.815
CK value U/L after CPFA	58.715	34.463	20.583	8.418
Myoglobin value ng/mL before CPFA	12.780	8.519	6.725	2.573
Myoglobin value ng/mL after CPFA	8.735	6.940	2.867	1.516

*Patient 3*				
*Blood flow *Qb, mL/min	*250*	*250*	*250*	*250*
Total plasma mL	*9.000*	*9.000*	*8.500*	*8.500*
Plasma filtration flow rate, QS%	*15*	*15*	*15*	*15*
Total dose of heparin I.U.	1.250	1.250	1.250	1.250
*Reinfusion solution *Qinf, mL/min	*35*	*35*	*35*	*35*
CK value U/L before CPFA	79.216	62.691	58.806	32.479
CK value U/L after CPFA	65.708	59.354	39.956	18.659
Myoglobin value ng/mL before CPFA	12.757	8.426	6.731	3.648
Myoglobin value ng/mL after CPFA	9.328	6.869	3.713	3.115

*Patient 4*				
*Blood flow *Qb, mL/min	*220*	*200*	*220*	
Total plasma mL	*7.500*	*8.000*	*7.200*	
Plasma filtration flow rate, QS%	*15*	*15*	*15*	
Total dose of heparin I.U.	1.250	1.250	1.250	
*Reinfusion solution *Qinf, mL/min	*35*	*35*	*35*	
CK value U/L before CPFA	30.389	16.495	11.699	
CK value U/L after CPFA	19.366	13.879	8.933	
Myoglobin value ng/mL before CPFA	9.562	3.815	1.318	
Myoglobin value ng/mL after CPFA	4.221	1.752	612	
